# Detoxifying symbionts in agriculturally important pest insects

**DOI:** 10.1111/1751-7915.12483

**Published:** 2016-12-12

**Authors:** Tijs J. M. van den Bosch, Cornelia U. Welte

**Affiliations:** ^1^ Department of MicrobiologyInstitute for Water and Wetland ResearchRadboud UniversityHeyendaalseweg 1356525AJ NijmegenThe Netherlands

## Abstract

Pest insects lead to excessive agricultural and therefore economical losses on crops worldwide. These insects have to withstand toxic molecules that are inherent to plant defences, as well as those that are produced and introduced by humans in the form of insecticides. In recent years, research on insect–microbe symbioses has recognized that microbial symbionts may play a role protecting against these toxins, leading to a form of defensive symbiosis between the pest insect and different types of microorganisms that we term detoxifying symbioses. In this minireview, we will highlight well‐characterized and emerging insect model systems of detoxifying symbioses and assess how the microorganisms influence the host's success.

## Introduction

Insects are very successful eukaryotic life forms on earth and have evolved into a stunning diversity of lineages. In agriculture, many insects are beneficial because they pollinate crops, disperse seeds or prey on herbivores (Dillon and Dillon, 2004). On the other hand, there are also detrimental insects that feed on crops and challenge food security worldwide, and are therefore regarded as pest insects. Less than 0.5% of the known species of insects are considered pests, yet several estimates of worldwide losses caused by insects indicate a staggering 7.9–15.1% of the world's annual crop production (Oliveira *et al*., 2014). To summarize which insect species are the most harmful is a futile task as the pest status is highly variable, dependent on year, weather, geography and insect biology and ecology. Furthermore, the monitoring and evaluation of damage caused by pest insects is poorly documented on a worldwide scale, and estimates of losses often have to be made based on limited data. With this in mind, we exemplify economic losses reported in Brazil in 2014, where greatest crop losses due to insects per area were observed for apples (4281 $/ha), tomatoes (3806 $/ha), tobacco (2729 $/ha), garlic (2655 $/ha), peanut (1679 $/ha), rubber (1242 $/ha) and grapes (1004 $/ha) (Oliveira *et al*., 2014).

There are several mechanisms known for counteracting pest insects, e.g. sterile insect techniques (SIT), crop rotation, chemical insecticides or biological pest control exploiting predators and parasitoids (Douglas, 2007). The former employs the release of male insects that are not able to produce fertile offspring, yet mate with female wild‐type insects, yielding a reduced amount of pest insects in the next generation. The use of chemical insecticides has been under debate in the past decades as many insecticides have harmful effects on humans (Costa *et al*., 2007), ecosystems and non‐target insects (Pimentel, 2005). Release of parasitoids is now a commercially available biocontrol strategy with varying success (Smith, 1996; Bokonon‐Ganta *et al*., 2013). A largely untapped resource that may be used in pest management is the manipulation of microorganisms that live in symbiosis with insects. It has been recognized for a long time that microorganisms play key roles in biogeochemical element cycles (Rousk and Bengtson, 2014), ecosystem functioning (Graham *et al*., 2016), host nutrition (Hacquard *et al*., 2015) and human health (Sun and Chang, 2014). Microbial symbionts provide an especially diverse range of benefits in insect nutrition, e.g. by providing essential amino acids (Douglas, 1998), digestive enzymes (Brune, 2014) or vitamins (Salem *et al*., 2014). One field that has received less attention is the roles that microbes play in protecting insects from toxic plant compounds and insecticides. This is despite the fact that it is known that many microorganisms contain enzymatic degradation mechanisms for a variety of plant secondary metabolites such as terpenes (Marmulla and Harder, 2014), caffeine (Summers *et al*., 2012, 2015), nicotine (Brandsch, 2006; Li *et al*., 2010), cocaine (Narasimhan *et al*., 2012), isothiocyanates (Fan *et al*., 2011) and even phosphorus‐ or sulfur‐containing insecticides (Kertesz *et al*., 1994). Oftentimes the interactions between microbe and insect are difficult to disentangle, and the relative contribution of insect versus microbial defence mechanisms is not yet known. In this minireview, we will focus on hitherto described pest insects where symbiotic microorganisms may play a crucial role in detoxifying detrimental compounds for their respective hosts, and touch upon possible routes for pest insect management strategies.

## Defining detoxifying symbiosis

The ecological definition of a symbiosis is broad, encompassing commensalism, parasitism and mutualism between any two dissimilar organisms. In insect–microbe interactions, the bacterial counterpart is generally referred to as ‘symbiont’ and the insect as ‘host’. As studies in microbe–insect interactions oftentimes also include the involved physiology of the plant that hosts an herbivorous insect, the plant may be referred to as ‘plant host’ or ‘host diet’. Symbionts can be distinguished as primary or secondary, which was particularly useful during the early days of research on the now well‐studied symbiosis between aphids and their primary symbionts *Buchnera aphidicola*. Primary symbionts that are important for proper functioning of the host are generally maternally transmitted and have remained unculturable even with modern culturing techniques. This fastidious nature is caused by drastic reduction in genome size (Moran and Bennett, 2014). Generally, this type of symbiosis, involving reduced symbiont genomes, is reserved for nutritional symbioses, but recent findings indicate that some primary symbionts may also have a defensive function in psyllids and stinkbugs (Hosokawa *et al*., 2006; Nakabachi *et al*., 2013). Primary symbionts are indispensable for both partners, resulting in perfect host infection rates. These symbioses, where essential nutrients are synthesized by an intracellular endosymbiont with a highly reduced genome, are particularly widespread in aphids, tsetse flies and psyllids (Douglas, 1998; Nikoh *et al*., 2011; Jing *et al*., 2014).

**Figure 1:**
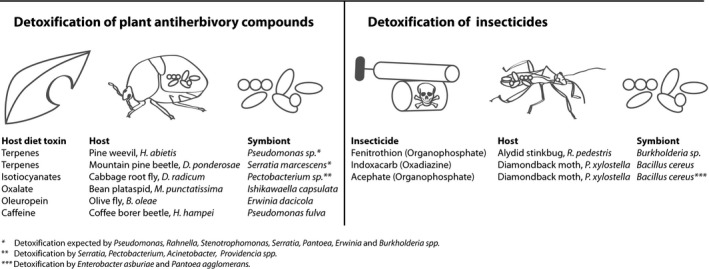
An overview of emerging models for symbiont‐mediated detoxification in pest insects.

Secondary symbionts are generally ubiquitous in many types of insects. They show less fidelity towards one specific host, and not all individuals of a population necessarily carry the symbiont. Loss of a secondary symbiont by an insect is a common occurrence, especially in lab‐reared cultures where the natural driving force behind a sustained symbiosis may be inevitably or inadvertently removed (Kellner, 1999; Rani *et al*., 2009; Estes *et al*., 2012). The biological effects of most facultative symbionts are unknown, but findings regarding their defensive capabilities imply that their roles will be understandable only in the context of complex natural environments (Moran *et al*., 2008). Examples of defensive symbionts conferring beneficial traits include, but are not limited to; increased thermal tolerance, production of antipredator toxins and protection against pathogens (Flórez *et al*., 2015). Many insect–microbe symbioses were recently extensively reviewed by several authors (Douglas, 2013, 2015; Hansen and Moran, 2014), but one aspect in particular has not yet been well evaluated, namely symbiont‐mediated detoxification (Fig. 1). As the definition of a defensive symbiosis does not fully capture the concept of symbiont‐mediated detoxification, we suggest the term ‘detoxifying symbiosis’ for this type of mechanism, where insect‐associated microorganisms are the main factor responsible for the detoxification of plant toxins or insecticides. It should be kept in mind that categories like ‘nutritional’, ‘defensive’ or ‘detoxifying’ symbiosis are not mutually exclusive for any given symbiosis and refer to only one elucidated or hypothesized aspect of a complex partnership. Recent developments in the field are providing increasingly more evidence for the natural occurrence of gut‐associated microorganisms that aid or possibly are crucial to detoxification. Here, we compile recent studies that indicate microbial components involved in the detoxification of toxic plant metabolites and man‐made insecticides (Adams *et al*., 2013; Ben‐Yosef *et al*., 2014; Ceja‐Navarro *et al*., 2015; Berasategui *et al*., 2016; Welte *et al*., 2016a).

## Detoxifying symbionts confer resistance to plant allelochemicals

As a result of their sessile lifestyle, plants have developed an impressive arsenal of defensive mechanisms, even when disregarding physical defences such as trichomes, prickles or thorns. They have evolved to produce a multitude of antiherbivorous compounds to deter feeding insects that we will simply refer to as allelochemicals. Many herbivorous insects specialize on one or just a few closely related host plants and thus have to overcome the defensive compounds that these plants produce. In many cases, defence against allelochemicals is an intrinsic part of insect physiology, mediated by insect‐produced enzymes like cytochrome P450 enzymes, glutathione S‐transferases or esterases (Douglas, 2015). In other cases, the mechanism with which an insect copes with the toxicity of its diet has remained unclear. In this chapter, we discuss studies that suggest that microbial symbionts aid their hosts in the detoxification of antiherbivorous compounds and supply limited economic context for these pest insects.

### Detoxifying symbionts of legume pests

Plataspids (or kudzu bugs, *Megacopta* spp.) are a notorious pest of peas, soya beans and other legumes, and received international notoriety after spreading from Asia to the United States in 2009 (Ruberson *et al*., 2012). Data on economic losses caused by the plataspid are scarce. However, occasional soya bean yield losses of up to 50% have been reported in China, and large infestations are now consistently found in the U.S.A., where smell and sheer number of plataspids can cause public annoyance (Ruberson *et al*., 2012). Plataspidae are known to vertically transmit their obligate *Candidatus* Ishikawaella capsulata symbiotic microbes by depositing brown symbiont‐containing capsules with their eggs that are ingested by newborn nymphs (Nikoh *et al*., 2011).

Sequencing of *Ca*. I. capsulata revealed an *ode* gene on one of its plasmids (Hosokawa *et al*., 2006; Nikoh *et al*., 2011). The *ode* gene codes for oxalate decarboxylase, which can break down oxalate; a plant secondary metabolite that provides defence against herbivory (Franceschi and Nakata, 2005). Soluble oxalic acid is a strong acid and is toxic to animals, while the crystallized calcium oxalate is reported to serve as a mechanical defence, as they have a striking abrasive effect on the mandibles of chewing insects (Korth *et al*., 2006). Feeding‐choice tests have shown that beet armyworms (*Spodoptera exigua*) avoid calcium oxalate containing wild‐type *Medicago truncatula* leaves in the presence of mutants that lack calcium oxalate. Breakdown of oxalate was already reported in gut bacteria found in humans, pigs and sheep (Allison *et al*., 1985). Future studies on the plataspid symbiont should consider the possibility of a similar detoxifying role for this insect. Nikoh and colleagues (Nikoh *et al*., 2011) thus suggested that *Ca*. I. capsulata may be giving not only nutritional but also protective benefits to its host.

*Ca*. I. capsulata shows remarkable resemblance to the *Buchnera* and *Wigglesworthia* genera that are found in obligate nutritional symbiosis with aphids and tsetse flies, respectively. A genetic analysis on the *Ca*. I. capsulata symbionts of pest plataspid *Megacopta punctatissima* revealed that the symbionts have a highly reduced genome (~746 kb) that contains genes involved in amino acid metabolism, indicative of obligate symbionts of sap‐feeding insects. Just like aphids, the stinkbug *M. punctatissima* feeds on phloem sap that contains little amino acids and its diet is supplemented by microbes that provide essential amino acids (Nikoh *et al*., 2011). It thus seems that *Buchnera* and *Ishikawaella* symbionts serve similar purposes for their respective hosts, but *Ca*. I. capsulata is not an endosymbiont. Instead, it can be found extracellularly in an enlarged portion of the posterior midgut of the adult stinkbug (Hosokawa *et al*., 2006). It is still considered an obligate symbiont because the experimental removal of *Ca*. I. capsulata from the insect caused retarded growth, a higher mortality rate and increased sterility, among other negative effects. Interestingly, when two species of plataspids with high and low pest status (*M. punctatissima* and *Megacopta cribraria,* respectively) had their obligate symbionts experimentally exchanged, their performances on a crop legume were also reversed (Hosokawa *et al*., 2007). This finding is particularly interesting with regard to possible insect control methods involving symbiotic microorganisms; if the pest status of an insect depends critically on symbiont genotype, this would provide a basis for identifying and/or selecting for genotypes geared towards specific pest management priorities. The next important (yet long outstanding) question is whether this type of selection is feasible with low‐tech management strategies like manipulation of the nutrition or abiotic factors experienced by insect pests (Douglas, 2007).

### Detoxifying symbionts of pine pests

Pine plantations are heavily impacted by mountain pine beetles (*Dendroctonus ponderosae*) and pine weevils (*Hylobius abietis*), two serious pests in the cultivation of pine seedlings and conifers causing losses of up to 80% (Petersson and Örlander, 2003; Boone *et al*., 2013). Beetles attempting to colonize living conifers will encounter a diverse array of toxic terpenes, produced by the tree for protection (Hamberger *et al*., 2011). Bacteria associated with *D. ponderosae* were first shown to be able to reduce concentrations of terpenes (Boone *et al*., 2013), and subsequent metagenomic sequencing of the microbiome of *D. ponderosae* revealed that its bacterial community is highly enriched in genes contributing to terpene metabolism (Adams *et al*., 2013). Recently, a study by Berasategui and co‐workers (Berasategui *et al*., 2016) found that the gut microbiome of pine weevils is also highly similar to closely related weevils and bark beetles feeding on similar food sources, raising the question whether those terpene‐degrading bacteria enable the pine weevil to overcome host plant defences (Berasategui *et al*., 2016). Similarly, a *Rhodococcus* species isolated from the generalist gypsy moth (*Lymantria dispar*) was closely related to the *Rhodococcus erythropolis* DCL14 that produces an enzyme responsible for monoterpene degradation (Broderick *et al*., 2004).

### Detoxifying symbionts of olive pests

The olive fly *Bactrocera oleae* is a major pest insect found on olive trees worldwide. In the Mediterranean basin, where 98% of olive trees are found, the pest has occurred for over 2000 years. In heavily infested regions, the olive fruit fly can cause crop losses of up to 100% for table olives and up to 80% for olive oil variants due to lower quality requirements (Zalom, 2009; Hamdan, 2016). Yearly economic losses caused by the olive fruit fly are estimated at 800 million $, despite a yearly investment of 100 million $ combating olive tree pests (El‐Hadi, 1996). Recent efforts for finding effective biocontrol strategies included screening for wild parasitoids in Himalayan Asia (Bon *et al*., 2016), and the braconid wasp *Psyttalia ponerophaga* shows favourable results in experimental setups (Sime *et al*., 2007). Currently, the primary control method employed by olive growers in California entails intensive spraying of spinosad insecticides (Vossen *et al*., 2004). Unripe olives are rich in oleuropein, a bitter tasting phenolic glycoside that is the main secondary metabolite of the unripe fruit. When activated by glycosylation, it cross‐links foliar proteins into high molecular weight aggregates and binds lysine residues, effectively reducing the nutritional value of dietary protein in the affected plant (Konno *et al*., 1999). Olive fly larvae have to overcome this defence strategy, and one possible mechanism is the association with symbiotic microorganisms. Several recent studies (Estes *et al*., 2009; Ben‐Yosef *et al*., 2014, 2015; Andongma *et al*., 2015) highlighted the possible involvement of bacteria even though they do not provide definitive proof of bacterial detoxification of the oleuropein secondary metabolite. When olive flies with and without (aposymbiotic) the bacterium *Ca*. Erwinia dacicola were reared on unripe olives, aposymbiotic larvae did not develop beyond the second instar. Conversely, oleuropein concentration is drastically lower in ripe olives, and aposymbiotic larvae were able to complete their development to adulthood on these fruits, although it took significantly longer and the larvae were significantly lighter than their symbiotic counterparts (Ben‐Yosef *et al*., 2015). Flies commonly associate with free‐living, rot‐inducing bacteria like those of the genus *Erwinia* that can be inoculated into the fruit by ovipositing females, subsequently enhancing larval nutrition with proteins and essential nutrients. These bacteria can usually grow on fruits independently of the host insect (Ben‐Yosef *et al*., 2015). The olive fly symbiont *Ca*. E. dacicola, however, has so far not been obtained in pure culture but has been studied by a variety of culture‐independent methods. The association of the olive fly with *Ca*. E. dacicola combines characteristics that were once thought to be exclusive for either obligate or facultative symbionts (Moran *et al*., 2008). Its genome size and GC‐content are not representative of classical primary endosymbionts and are more indicative of free‐living or phytopathogenic bacteria. On the other hand, *Ca*. E. dacicola is vertically transmitted and shows a clear history of co‐evolution with its host like classical primary endosymbionts (Capuzzo *et al*., 2005). *Ca*. E. dacicola was found in all life stages and in all wild populations of *B. oleae* in both the New and Old World sampled over 2 years with a significant difference in relative abundance across life stages (Estes *et al*., 2012). Abundances were highest in ovipositing females and low in eggs and pupae (Estes *et al*., 2009). It was hypothesized that the high abundance of symbiotic *Ca* E. dacicola in ovipositing females causes a higher chance of vertical transmission to progeny and may possibly provide dietary supplementation during the nutritionally demanding period of oviposition. The association of *Ca*. E. dacicola with all olive fly life stages suggests that these bacteria can persist through metamorphosis and dietary changes. About 10% of the adult population appeared to lack the bacterium, so even though *Ca*. E. dacicola is tightly associated with the olive fly it may either not be obligate in terms of host survival or was present below the detection limit of the chosen method. Larvae void their intestine prior to pupation and might therefore contain a very low amount of symbiotic gut bacteria, leading to too low amounts of template DNA to detect the symbiont with 16S rRNA gene targeting methods (Estes *et al*., 2009). *Ca*. E. dadicola is maintained extracellularly in the adult host microbiome in the gut lumen, the gastric caeca (Ben‐Yosef *et al*., 2015) and intracellularly in the midgut epithelial cells of larvae (Estes *et al*., 2009). A significant nutritional role has been demonstrated for this bacterium, as it provides the capacity to utilize non‐essential amino acids and urea as a nitrogen source (Ben‐Yosef *et al*., 2014). The studies have indicated that it enables the olive fly to overcome plant defences, and it is implied that detoxification of plant oleuropein constitutes a major part in this defensive mechanism. Whether *Ca*. E. dacicola is the responsible element for the detoxification of oleuropein should be further investigated. The exact mechanism remains elusive and is difficult to validate without cultivation of *Ca*. E. dacicola, but hypotheses include symbiotic excretion of proteins that degrade, bind, or are resistant to polyphenols. The study further emphasizes the added value of being able to isolate a symbiotic bacterium for experimental purposes, which is still a major hurdle in many symbiotic models.

### Detoxifying symbionts of cabbage pests

Another fly species that may make use of detoxifying bacterial symbionts is the cabbage root fly, *Delia radicum*. It feeds on plants of the Brassicaceae family to which many well‐known vegetable crops belong like cabbages, cauliflower or brussels sprouts, as well as oilseed rape used for the production of vegetable oil and biodiesel. Although exact numbers are scarce, economical losses inflicted by *D. radicum* include crop losses of up to 80% but are highly dependent on the host plant and crop rotation. Yield reduction in three consecutive years of canola production, for example, amounted to economic losses of approximately 292–377 Can$/ha/year, mainly due to *D. radicum* infestation (Dosdall *et al*., 2012). Insecticide treatments using the organophosphate insecticide chlorpyrifos can be effective, but are banned in several countries. Cruciferous plants employ a common defence mechanism where toxic isothiocyanates (ITC) are produced by the breakdown of glucosinolates as catalysed by the plant enzyme myrosinase, as a response to insect damage. Most insect herbivores that specialize on glucosinolate‐containing plants avoid the production of ITC altogether (Winde and Wittstock, 2011). Infestation of the cabbage root fly, however, does lead to the emittance of ITC by plant roots (Crespo *et al*., 2012). Cabbage root fly larvae appear to lack any of the known avoidance mechanisms observed in other cabbage pests, such as phloem sucking to avoid glucosinolate disruption or enzymatically guided glucosinolate breakdown towards less toxic epithionitriles as an end product instead of ITC (Wittstock *et al*., 2003). A recent study shows that cabbage root fly larvae harbour gut bacteria capable of detoxifying the ITC themselves (Welte *et al*., 2016a). Four Gammaproteobacterial isolates (*Serratia*,* Providencia*,* Pectobacterium* and *Acinetobacter*) from the gut were able to degrade the ITC to less harmful compounds. Genome sequencing of these four isolates results in the assembly of several near‐identical plasmids recurring in the different genera, suggesting horizontal gene transfer. More specifically, the presence of *tra* genes indicates the occurrence of conjugation events. One plasmid, designated as Drgb3, contains the gene *saxA* (Fan *et al*., 2011) that encodes a novel ITC hydrolase (Welte *et al*., 2016b) which hydrolyses several aromatic ITC. The respective bacterial species were dominant players in the gut microbial community as judged by a metagenome analysis, and may therefore reduce the levels of toxic isothiocyanate in the host gut, benefiting host fitness.

### Detoxifying symbionts in coffee pests

The coffee borer beetle or coffee berry borer (*Hypothenemus hampei*) is the primary pest for coffee bean farmers across the world. Over 20 million coffee‐growing families are economically affected by this pest, and infestation levels are particularly high in plantations in Tanzania (90%), Malaysia (50–90%), Uganda (80%), Colombia (60 %), Mexico (60%) and Jamaica (58–85%; Jaramillo *et al*., 2006). Yearly losses have been estimated at 500 million $ worldwide, but accumulative approximations of economic loss per country indicate that this is a very conservative estimate (Vega *et al*., 2015). Caffeine is an important substance that gives coffee its stimulating effects, but in an ecological context coffee beans use caffeine as a protective alkaloid allelochemical against herbivory. The coffee borer beetle overcomes the toxicity of caffeine using its gut microbes for caffeine degradation as demonstrated by Ceja‐Navarro *et al*. (2015). Beetles with an intact gut flora were able to fully deplete the caffeine in their diet, whereas beetles that had their gut flora disrupted with an antibiotic treatment lost their ability to degrade caffeine. Interestingly, the researchers were able to reinstate the caffeine degradation in *H. hampei* by transferring a pure culture of *Pseudomonas fulva* into the gut via inoculum of the beetles’ artificial diet. This *P. fulva* had beforehand been isolated from the digestive tract of *H. hampei* and showed strong caffeine degrading capabilities of its own. Although many of the core microorganisms from *H. hampei* were able to subsist on caffeine, only *P. fulva* isolates yielded positive amplification of the *ndmA* gene which codes for an enzyme that catalyses the first step in caffeine degradation (Summers *et al*., 2012). This gene was also expressed *in situ,* further implying the importance of the role of *P. fulva* in detoxifying caffeine for its host.

The study of Ceja‐Navarro *et al*. provides excellent insight into the importance of bacteria in the detoxification of plant secondary metabolites. However, like many studies in this field, it also raises several new questions. For instance, out of over 100 isolates over 12 different bacterial species that were able to subsist on caffeine as their sole carbon and nitrogen source, only *P. fulva* contained the *ndmA* gene that is associated with caffeine breakdown. What are the metabolic pathways of the others? And even though infecting *H. hampei* with pure *P. fulva* was enough to reinstate caffeine degradation, the abundance of other species in natural core microbiomes suggests additional unknown functions for these bacteria.

## Symbiont‐mediated insecticide resistance

Plant‐produced toxins are not the only dangerous chemicals that insects encounter. Insecticides are employed to increase crop yields and control hygienic pests, contributing to worldwide agriculture, economy and health (Kikuchi *et al*., 2012). The rapid development of insecticide resistance by diverse organisms has raised concern and should be further investigated. Multiple mechanisms for insecticide resistance have been attributed to host‐level physiology (Kasai *et al*., 2000; Temeyer *et al*., 2008; Tabashnik and Carriere, 2010), but in recent years, some scientists argue that certain insecticide resistances may be attributable to detoxifying symbionts. Isolates of insect fungal symbionts have long been shown to be promising sources of detoxifying enzymes that target toxic allelochemicals as well as insecticides (Shen and Dowd, 1991; Dowd, 1992; Shen and Dowd, 1992). Evidence of insecticide resistance conferred by symbiont‐level physiology is still scarce, but particularly convincing results regarding a notorious Asian legume pest were recently put forward and will be discussed in the next paragraph.

### Insecticide resistance in the Japanese legume pest *Riptortus pedestris*

The broad‐headed bugs of the Alydidae family, particularly *Riptortus* spp. and *Leptocorisa* spp., are major pests on soya beans and other legumes in parts of eastern Asia, including Japan. *Riptortus clavatus* houses a dense population of *Burkholderia* symbionts in their midgut crypts, which are acquired from the environment in every generation, rather than the ‘conventional’ method of vertical maternal transmission (Kikuchi *et al*., 2007). The symbiotic *Burkholderia* are able to degrade the insecticide fenitrothion, a prevalent organophosphorus agent in agriculture (Kikuchi *et al*., 2012). Moreover, individual bugs of *Riptortus pedestris* readily establish a symbiosis with fenitrothion‐degrading *Burkholderia* symbionts and subsequently show much higher survival rates on plants dipped in fenitrothion than bugs with non‐degrading *Burkholderia* symbionts (Kikuchi *et al*., 2012). Application of fenitrothion to a field results in a relative increase of fenitrothion‐degrading bacteria in the soil, which is hypothesized to affect the dynamics of transmission of symbiotic degrading *Burkholderia* from the soil to stinkbugs (Tago *et al*., 2015). These findings suggest that insecticide resistance might develop in a field even in the absence of pest insects, which could then quickly establish in a single insect generation (Kikuchi *et al*., 2012). The established *Riptortus pedestris* model provides a good opportunity for studying bacterial symbiotic factors at a molecular level, as the *Burkholderia* symbionts are cultivable and genetically manipulable (Kim *et al*., 2015). Development of an ecological insecticide by utilizing gut symbionts is one of the long‐term research goals in the field. The studies regarding fenitrothion may only represent the proverbial tip of the iceberg and could thus be applicable to many as of yet undiscovered mechanisms of insecticide resistance.

### Insecticide breakdown by diamondback moth‐associated symbionts

The diamondback moth *Plutella xylostella* (sometimes also called the cabbage moth, not to be confused with *Mamestra brassicae*) is a major global pest of cruciferous crops, estimated to cost approximately 4 billion euros per year in lost production and management (Zalucki *et al*., 2012). Unlike the aforementioned *D. radicum*,* P. xyolostella* produces an enzyme that prevents formation of dietary isothiocyanates (Ratzka *et al*., 2002). *P. xylostella* is not only able to overcome host defences, but it has even been shown to be highly resistant to a large variety of chemical insecticides and it is one of the only three insect species to have developed resistance to *Bacillus thuringiensis*‐based insect control methods (Furlong *et al*., 2013). The rapid development of highly resistant phenotypes of *P. xylostella* is at least in part attributed to the insect's own physiology, and includes altered target sites for carbamates and organophosphates, metabolism of parathion via glutathione S‐transferases and detoxification of pyrethroids via microsomal P‐450 monooxygenases (Ramya *et al*., 2016). Isolated *Bacillus cereus* colonizing the moth's gut were able to break down the insecticide indoxacarb for use in metabolism and growth (Janmaat and Myers, 2003; Ramya *et al*., 2015). Another insecticide, acephate, was also readily broken down by bacteria isolated from the gut of the diamondback moth. These findings, combined with findings on symbiont‐mediated insecticide resistance in stinkbugs (Kikuchi *et al*., 2012), indicate that bacteria may play a larger role in insect resistance to insecticides than previously thought. However, *in vivo* breakdown of either insecticide by *P. xylostella* gut bacteria has not yet been demonstrated, so cause and effect remain unclear. Even if detoxifying symbiosis does not play a role in this association, further study can still be fruitful because organophosphorus‐degrading bacteria by themselves may prove of value in ecological and industrial applications (for an overview we refer to the review of Singh, 2009).

## Conclusions and outlook

In agriculturally important pest insects, the microbiome offers potential for improvement of current methods in pest management. Primary opportunities include the prediction of host traits and thereby the efficacy of control strategies, targeting of the microbiome for direct pest control or targeting the microbiome to reduce the vector competence of the pest (Douglas, 2015). Even though many studies speculate on the contribution of microorganisms in detoxifying symbiosis it is – even with the current advances in culture‐independent microbiological techniques – still challenging to unravel specific contributions of microbial players in the complete metabolism. Many detoxifying symbioses are specific to a certain type of insect lifestyle, including the specialization to a host plant producing toxic allelochemicals. Microbes are known to break down a large array of allelochemicals and insecticides which offers many opportunities for insects to establish detoxifying symbioses. Evolution in microorganisms proceeds at a faster pace than in insects, which might lead to quick adaptation of pest insects to insecticides by the use of symbiotic microorganisms (Kikuchi *et al*., 2012). Insects are furthermore able to rapidly acquire novel metabolic functions and to invade new ecological niches by engaging in symbiotic relationships with microbes that already possess complete, well‐attuned metabolic pathways (Hosokawa *et al*., 2016).

An increased demand for novel insect pest management created by growing human populations and global climate change is anticipated, and symbiotic microorganisms offer one potential route to meet this demand (Douglas, 2007). Of the symbiont‐based pest control strategies, only sterile insect technique is currently routinely used, and developments in paratransgenesis are ongoing but require genetic modification, making it difficult to apply in agricultural systems. Further research efforts into (detoxifying) symbiosis may lead to environmentally friendly and sustainable methods to control major pest insect populations. For example, if insect pest status depends critically on symbiont genotype, it would provide a basis for identifying and/or selecting for genotypes geared towards specific pest management priorities, ideally using low‐tech management strategies. Isolation of detoxifying symbionts could lead to applications in bioremediation or treatments for insecticide poisonings. In this minireview, we have assembled relevant studies regarding detoxifying symbioses in agriculturally important pest insects and how they were investigated, thereby providing an array of experimental strategies to learn more about microbes and their role in detoxifying symbiosis.

## Conflict of interest

The authors have no conflict of interest to declare.
